# Increasing Drug Resistance in Extensively Drug-Resistant Tuberculosis, South Africa

**DOI:** 10.3201/eid1703.101363

**Published:** 2011-03

**Authors:** N. Sarita Shah, Jessica Richardson, Prashini Moodley, Salona Moodley, Palav Babaria, Melissa Ramtahal, Scott K. Heysell, Xuan Li, Anthony P. Moll, Gerald Friedland, A. Willem Sturm, Neel R. Gandhi

**Affiliations:** Author affiliations: Tugela Ferry Care and Research Collaboration, Tugela Ferry, South Africa (N.S. Shah, J. Richardson, P. Babaria, S.K. Heysell, A.P. Moll, G. Friedland, N.R. Gandhi);; Albert Einstein College of Medicine, Bronx, New York, USA (N.S. Shah, J. Richardson, X. Li, N.R. Gandhi);; Montefiore Medical Center, Bronx (N.S. Shah, J. Richardson, X. Li, N.R. Gandhi);; Nelson R. Mandela School of Medicine, Durban, South Africa (P. Moodley, S. Moodley, M. Ramtahal, A.W. Sturm);; Yale University, New Haven, Connecticut, USA (P. Babaria, G. Friedland);; Philanjalo Care Center, Tugela Ferry (A.P. Moll)

**Keywords:** Extensively drug-resistant tuberculosis, tuberculosis and other mycobacteria, HIV, drug resistance, second-line drugs, South Africa, dispatch

## Abstract

We expanded second-line tuberculosis (TB) drug susceptibility testing for extensively drug-resistant *Mycobacterium tuberculosis* isolates from South Africa. Of 19 patients with extensively drug-resistant TB identified during February 2008–April 2009, 13 (68%) had isolates resistant to all 8 drugs tested. This resistance leaves no effective treatment with available drugs in South Africa.

Extensively drug-resistant tuberculosis (XDR TB) was first reported in 2005 and has been identified worldwide (*1**,**2*). XDR TB is associated with poor treatment outcomes (*3*), especially among persons co-infected with HIV (*4**,**5*). XDR TB strains are created when multidrug-resistant TB (MDR TB) is inadequately treated, which enables amplification of second-line drug resistance (*6**,**7*). Inadequate treatment for XDR TB may result in additional resistance, severely limiting options for effective treatment.

Drug-susceptibility testing (DST) for first-line and second-line TB drugs is essential for developing effective MDR TB and XDR TB treatment regimens (*8**,**9*). However, DST requires laboratory facilities that are unavailable in most settings with high incidence of TB. In the last global report of drug resistance, only one third of countries conducted routine DST on suspected cases of TB; the remaining countries restricted testing to high-risk patients (*2*). Thus, most high-incidence settings use standardized MDR TB or XDR TB treatment regimens based on epidemiologic data from periodic drug-resistance surveys. Because the epidemiology of drug-resistant TB is changing rapidly (*10*), successful use of standardized regimens depends on accurate population-level drug-resistance data.

In 2005, a large HIV-associated XDR TB epidemic was detected in Tugela Ferry, South Africa (*5*). Continuous, routine drug resistance surveillance for all suspected TB cases has since been implemented in Tugela Ferry and >500 XDR TB cases have been diagnosed; >90% of patients are coinfected with HIV. Most early XDR TB isolates from Tugela Ferry were resistant to 4 or 5 drugs (isoniazid, rifampin, ofloxacin, and kanamycin, plus ethambutol or streptomycin) (*4*). XDR TB isolates have become resistant to an increasing number of drugs, such that by 2007, >90% were resistant to all 6 first-line and second-line drugs tested (Figure 1) (*4*).

**Figure 1:**
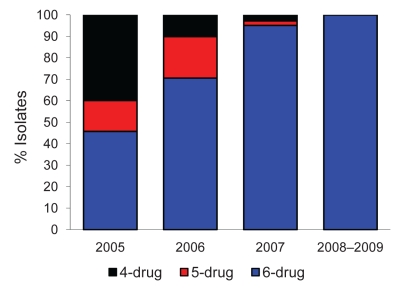
Drug resistance among extensively drug-resistant tuberculosis isolates from Tugela Ferry, South Africa, 2005–2009: 4-drug resistance = isoniazid (INH), rifampin (RIF), ofloxacin (OFL), and kanamycin (KM); 5-drug resistance = INH, RIF, OFL, KM, and ethambutol (EMB) or streptomycin (SM); 6-drug resistance = INH, RIF, OFL, KM, EMB, and SM. Column for 2008–2009 indicates study population.

DST for other second-line drugs (in addition to ofloxacin and kanamycin) is not routinely performed in South Africa. However, the standardized XDR TB treatment regimen includes second-line drugs for which drug resistance data are lacking. Thus, we sought to further characterize second-line TB drug resistance among XDR TB isolates in Tugela Ferry by expanding DST to include capreomycin and ethionamide.

## The Study

This study was conducted in Tugela Ferry, South Africa, where TB incidence is ≈1,100 cases/100,000 population and >80% of TB case-patients are HIV infected. MDR TB and XDR TB incidence was 118 cases and 72 cases/100,000 population, respectively, in 2007 (*4*). Ethical approval for this study was obtained from Albert Einstein College of Medicine, Yale University, University of KwaZulu-Natal, and the KwaZulu-Natal Department of Health.

We performed a prospective cross-sectional study actively identifying patients with suspected TB in medical and TB wards, the HIV clinic, and the outpatient department at the Tugela Ferry district hospital during February 2008–April 2009. A person with suspected TB was defined as someone having a self-reported cough of any duration or >2 other signs or symptoms, including fever, night sweats, weight loss, or shortness of breath for any duration. Patients could be either newly manifesting TB symptoms or have been receiving TB treatment for >2 months but currently reporting active TB symptoms (i.e., treatment failures). One sputum specimen was obtained for culture and DST.

Sputum for this study was tested by microscopic analysis of auramine- and Ziehl-Nielsen–stained smears and Middlebrook 7H11 agar and Mycobacterial Growth Indicator Tube 960 broth culture. Identification of *Mycobacterium tuberculosis* was confirmed by using niacin and nitrate reductase tests. DST of positive cultures was performed by using the 1% proportional method on Middlebrook 7H11 agar for isoniazid (critical concentrations: isoniazid 0.2 µg/mL, rifampin 1.0 µg/mL, ethambutol 7.5 µg/mL, streptomycin 2.0 µg/mL, ofloxacin 2 µg/mL, kanamycin 5.0 µg/mL, capreomycin 10 µg/mL, and ethionamide 5.0 µg/mL). DST was repeated on all drug-resistant isolates to confirm the observed resistance pattern.

Medical records were reviewed for demographic and clinical data. The proportion of patients with XDR TB and drug-susceptibility patterns were described by using simple frequencies. XDR TB treatment outcomes were reported as of November 2009; standard international definitions were used (*11*).

**Figure 2:**
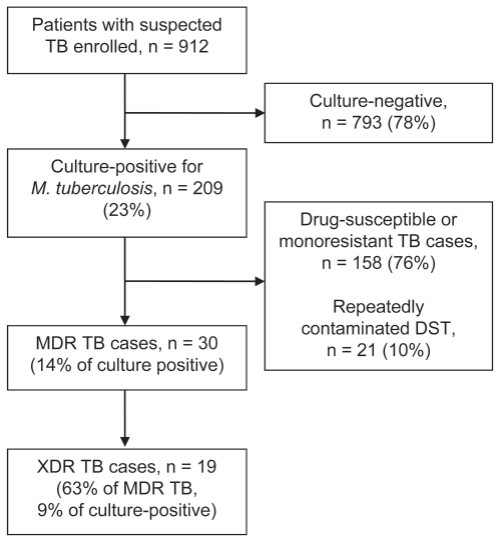
Determination of prevalence of tuberculosis (TB) and drug resistance among persons with suspected TB, Tugela Ferry, South Afica, 2008–2009. DST, drug susceptibility testing; MDR TB, multidrug-resistant TB; XDR TB, extensively drug-resistant TB.

Of 912 enrolled patients with suspected TB, 209 (23%) had culture-positive TB (Figure 2). Of these patients, 30 (14%) had MDR TB, of which 19 (63% of those with MDR TB; 9% with culture-positive results) had XDR TB.

Among XDR TB isolates, all 19 (100%) were resistant to all 6 drugs routinely tested in KwaZulu-Natal Province (isoniazid, rifampin, ethambutol, streptomycin, ofloxacin, and kanamycin), which extended the trend seen in previous years toward increasing drug resistance (Figure 1). Of these isolates, 4 (21%) were also resistant to capreomycin, and 13 (68%) were resistant to capreomycin and ethionamide (Table 1). Thus, an 8-drug resistance pattern was the predominant DST type among XDR TB patients in this cohort.

**Table 1:** Drug susceptibility test results for 19 XDR TB patients, South Africa*

Drug resistance pattern (antibiogram)	No. (%) patients
INH, RIF, EMB, SM, OFL, KM	2 (11)
INH, RIF, EMB, SM, OFL, KM, CAP	4 (21)
INH, RIF, EMB, SM, OFL, KM, CAP, ETO	13 (68)
Total	19 (100)

*XDR TB, extensively drug-resistant tuberculosis; INH, isoniazid; RIF, rifampin; EMB, ethambutol; SM, streptomycin; OFL, ofloxacin; KM, kanamycin; CAP, capreomycin; ETO, ethionamide.

Of 13 patients with 8-drug resistance XDR TB, 5 (38%) were women (median age 33.5 years, range 24–51 years) (Table 2). Although 5 (38%) had previously received (or currently showed failure to) first-line TB treatment, none had ever received treatment with second-line drugs for MDR TB. Seven (54%) patients were identified from the ambulatory HIV clinic. Twelve (92%) patients were HIV infected (median CD4 cell count 183.5 cells/mm^3^, range 22–670 cells/mm^3^); only 2 (17%) were receiving antiretroviral therapy at the time of TB screening.

**Table 2:** Characteristics of 30 MDR TB and XDR TB patients, South Africa*

Characteristic	MDR TB	6- or 7-drug XDR TB	8-drug XDR TB
Total	11	6	13
Female sex	8 (73)	4 (67)	5 (38)
Age, y, median (range)	36 (26–52)	42.5 (36–64)	33.5 (24–51)
Prior TB treatment			
First-line drugs†	5 (45)	5 (83)	5 (38)
Second-line drugs‡	0	0	0
TB contact	2 (18)	2 (33)	1 (8)
Enrollment site at HIV clinic	8 (73)	2 (33)	7 (54)
HIV positive	10 (91)	5 (83)	12 (92)
CD4 cell count, cells/mm^3^, median (range)	155 (25–708)	117.5 (18–426)	183.5 (22–670)
Receiving antiretroviral therapy (among HIV-positive patients)	7 (70)	2 (40)	2 (17)

*Values are no. (%) unless otherwise indicated. MDR TB, multidrug-resistant tuberculosis; XDR TB, extensively drug-resistant TB. 6-drug resistance, resistance to isoniazid, rifampin, ethambutol, ofloxacin, kanamycin, and streptomycin; 7-drug resistance, resistance to isoniazid, rifampin, ethambutol, ofloxacin, kanamycin, streptomycin, and capreomycin; 8-drug resistance, resistance to isoniazid, rifampin, ethambutol, ofloxacin, kanamycin, streptomycin, capreomycin, and ethionamide. †First-line drugs used for treatment of persons with new TB cases or confirmed drug-susceptible TB include isoniazid, rifampin, ethambutol, and pyrazinamide. ‡Second-line drugs used for treatment of persons with confirmed MDR TB include ofloxacin, kanaymycin, ethionamide, *p*-aminosalicylic acid, and cycloserine or terizidone.

Among 13 XDR TB patients with 8-drug resistance, 7 (54%) died (median time to death 59 days, range 16–205 days). Two patients were lost to follow-up, and 4 (31%) are still living and receiving XDR TB treatment (range 190–502 days of follow-up). No trend in survival of patients with XDR TB was observed by drug-resistance pattern (6-drug vs 7-drug vs. 8-drug).

## Conclusions

Routine drug-resistance surveillance to first- and second-line drugs is conducted in Tugela Ferry, which has a high incidence of TB and HIV co-infection. In this study, we expanded second-line testing for 2 additional bactericidal drugs (capreomycin and ethionamide) for treatment of patients with XDR TB. Resistance to 8 first-line and second-line drugs is the predominant pattern for XDR TB in Tugela Ferry, thereby severely limiting effective therapeutic options with available medications. According to the standard XDR TB regimen used in this province, patients were receiving <3 active drugs (pyrazinamide, *p*-aminosalicylic acid, and cycloserine), which increases the risk for treatment failure and further amplification of drug resistance. These findings underscore the need for routine surveillance for resistance to all first-line and second-line drugs used and for tailoring regimens accordingly to improve treatment success and reduce emergence of more drug-resistant XDR TB strains.

This study had 3 main limitations. First, the reliability of second-line DST is variable, and only recently have methods and critical concentrations been standardized (*12*). However, all drug-resistant isolates in this study had DST repeated to confirm observed results. Second, DST for other first-line drugs, such as pyrazinamide, and other second-line drugs was not conducted, although these drugs are often used for XDR TB treatment. Thus, the degree of drug resistance was likely to be only a minimum estimate. Third, although the proportion of XDR TB cases in this survey was high, the absolute number of XDR TB cases was low. This small sample size limits our ability to make conclusions about treatment outcomes for patients with increasing drug-resistant isolates. However, previous studies from our site have shown poorer survival rates with increasing drug resistance (*4*).

Expanded DST for second-line and third-line drugs is critical for XDR TB patient care. Given continued high and rapid number of deaths from XDR TB, better and more rapid methods for second-line DST are urgently needed to improve diagnosis and guide treatment. Although new drugs are being developed, efforts must target prevention of XDR TB and its transmission, earlier identification of cases, support of treatment completion for TB and MDR TB, and greater use of antiretroviral therapy for patients who are co-infected with HIV.
